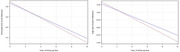# Association Between Mean Corpuscular Volume and Cognitive Function in Community‐Dwelling Older Adults ‐ An 11‐year Cohort Study

**DOI:** 10.1002/alz.088659

**Published:** 2025-01-09

**Authors:** Pei‐Yu Yen, Jen‐Ming Chiou, Yen‐Ching Chen, Jen‐Hau Chen

**Affiliations:** ^1^ Master of Public Health Degree Program, College of Public Health, National Taiwan University, Taipei Taiwan; ^2^ Taipei City Hospital, Taipei Taiwan; ^3^ Institute of Statistics and Data Science, National Taiwan University, Taipei Taiwan; ^4^ Institute of Epidemiology and Preventive Medicine, College of Public Health, National Taiwan University, Taipei Taiwan; ^5^ Department of Public Health, College of Public Health, National Taiwan University, No. 17, Xu‐Zhou Road, Taipei Taiwan; ^6^ Department of Geriatrics and Gerontology, National Taiwan University Hospital, Taipei Taiwan

## Abstract

**Background:**

The mean corpuscular volume (MCV) is a common parameter in routine blood tests. Larger MCV tends to be more fragile and pose challenges in passing through capillaries, leading to a diminished capacity for oxygen and nutrient supply to the brain thus impacting cognitive function. Limited studies have explored the association between MCV and cognitive impairment with inconsistent results. This study aimed to investigate the association between MCV and cognitive function in community‐dwelling older adults.

**Method:**

This 11‐year cohort study (2011‐2022) included 530 non‐demented older adults (aged above 65) from the ongoing Taiwan Initiative for Geriatric Epidemiological Research at baseline (2011 to 2013) with four biennial follow‐ups. Global cognition was assessed by the Taiwanese version of the Montreal Cognitive Assessment. Domain‐specific cognition, including memory, attention, verbal fluency, and executive function, was assessed by a battery of neuropsychiatric tests. Blood data were collected, including MCV, red blood cell distribution width, hemoglobin, platelet, and vitamin B12. The cutoff of MCV was determined by the turning point obtained from the generalized additive model. This study utilized a generalized linear mixed model to analyze the association between MCV and cognitive function adjusted for age, sex, years of education, apolipoprotein E ε4 status, years of follow‐up, and other blood parameters.

**Result:**

The average age of the participants is 72.68 years, with females comprising 53.40%. As the follow‐up time increased, older adults with larger baseline MCV (>93.0 fL) exhibited a significantly poor performance of memory (immediate free recall: = ‐0.028, p = 0.021; delayed free recall: = ‐0.039, p = 0.002; delayed theme recall: = ‐0.041, p = 0.003), and attention (digit span forward: = ‐0.028, p = 0.027). Additionally, an interaction was found between MCV and age on global cognition (p _interaction_=0.008).

**Conclusion:**

Over time, increased MCV was associated with poor domain‐specific cognition. MCV may potentially serve as an early indicator for assessing cognitive impairment. Larger and longer‐term studies are needed to clarify their temporal relationship and underlying mechanism.